# Application of Time-Domain Full Waveform Inversion to Cross-Hole Radar Data Measured at Xiuyan Jade Mine, China

**DOI:** 10.3390/s18093114

**Published:** 2018-09-15

**Authors:** Sixin Liu, Xintong Liu, Xu Meng, Lei Fu, Qi Lu, Li Deng

**Affiliations:** 1 College of Geo-Exploration Science and Technology, Jilin University, Changchun 130026, China; phd_liuxintong@hotmail.com (X.L.); fuleijlu@163.com (L.F); luqi@jlu.edu.cn (Q.L.); 2 State Key Laboratory of Lunar and Planetary Sciences, Macau University of Science and Technology, Macau, China

**Keywords:** cross-hole radar tomography, FWI, conductivity, permittivity, mined-out zone

## Abstract

Xiuyan Jade, produced in Xiuyan County, Liaoning Province, China is one of the four famous jade in China. King Jade, which is deemed the largest jade body of the world, was broken out from a hill. The local government planned to build a tourism site based on the jade culture there. The purpose of the investigation was to evaluate the stability of subsurface foundation, and the possible positions of mined-out zones to prevent the further rolling of the jade body. Cross-hole radar tomography is the key technique in the investigation. Conventional travel time and attenuation tomography based on ray tracing theory cannot provide high-resolution images because only a fraction of the measured information is used in the inversion. Full-waveform inversion (FWI) can provide high-resolution permittivity and conductivity images because it utilizes all the information provided by the radar signals. We deduce the gradient expression of the time-domain FWI with respect to the permittivity and conductivity using a method that is different from that of the previous work and realize the FWI algorithm that can simultaneously update the permittivity and conductivity by using the conjugate gradient method. Inverted results from synthetic data show that time-domain FWI can significantly improve the resolution compared with the ray-based tomogram methods. FWI can distinguish targets that are as small as one-half to one-third wavelength and the inverted physical values are closer to the real ones than those provided by the ray tracing method. We use the FWI algorithm to the field data measured at Xiuyan jade mine. Both the inverted permittivity and conductivity can comparably delineate four mined-out zones, which exhibit low-permittivity and low-conductivity characteristics. Furthermore, the locations of the interpreted mined-out zones are in good agreement with the existing mining channels recorded by geological data.

## 1. Introduction

Ground penetrating radar (GPR) has been widely used in environmental, hydrological and engineering geophysics due to its capability of delineating the complex near-surface structure [1]. The cross-hole radar, with its transmitting and receiving antennas (with dominant frequencies of 20–250 MHz) located in two adjacent holes, acquires the radar wave which can be used to deduce geological information by inverting the subsurface permittivity and conductivity spatially.

Traditional cross-hole radar imaging methods are based on ray tracing theory, such as travel time inversion and maximum amplitude of the first cycle signal inversion [2,3,4,5]. The combination of travel time tomography and migration is introduced [6]. A smoothed velocity model obtained by travel time tomography is used as the basis of migration. Then, a reverse-time migration technique is applied to cross-hole data after direct-wave suppressing. Due to the high-frequency assumption in the ray-based method, the resolution of these methods is around the diameter of first Fresnel zone [7].

In order to improve the resolution, methods based on full waveform inversion (FWI) techniques have been introduced for seismic imaging formerly. Tarantola had carried out the pioneering works [8,9,10]. The nonlinear inverse problem for seismic reflection data is solved in the acoustic approximation. A method based on the generalized least-squares criterion was introduced, and it could handle errors in the data set and priori information on the model [8]. A strategy for nonlinear elastic inversion of seismic reflection data was suggested for separation of the parameters describing the Earth in long spatial wavelengths and short spatial wavelengths [10]. Later, many works have been done for FWI in frequency domain [11,12,13]. A comparison has been taken for Gauss-Newton and full Newton methods in frequency-space seismic waveform inversion [11].

FWI was adapted from seismic data processing to cross-hole radar data processing. Kuroda et al. [14] and Ernst et al. [15,16] used the FWI method for cross-hole radar data and got good results formerly. The difference is that Kuroda et al. [14] only inverted the permittivity but Ernst et al. [15,16] used the cascade method to update the permittivity and conductivity simultaneously. Meles et al. [17] proposed a vector FWI technique to simultaneously invert the permittivity and conductivity. Belina et al. [18] analyzed the source wavelet effect on the FWI. Wu et al. [19] presented the detailed derivation of the theoretical foundation of the FWI and used the steepest descent method to update the model parameters.

The cross-hole radar FWI technique has been developed and applied widely in hydrogeology. Klotzsche et al. used the FWI algorithm to invert cross-hole GPR data acquired within a gravel aquifer in the northern Switzerland and obtained significantly high-resolution images [20,21]. Their research shows that FWI of cross-hole radar signals could image a low-velocity wave-guiding layer within subwavelength thickness. The waveguide trapping causes anomalously high amplitudes and elongated wave trains. Klotzsche et al. [22,23] determined the lateral extent of low-velocity waveguides using a pseudo-3D FWI of six cross-hole GPR cross-sections within a square configuration of four boreholes. Yang et al. [24] introduced normalized gradients that do not depend on the number of sources and receivers which enable a comparison of the gradients and step increments for different cross-hole survey layouts. Nonetheless, cross-hole radar survey is time-consuming in data acquisition and expensive in computational costs. To reduce these expenses, a semi-reciprocal acquisition setup with a reduced number of transmitters and an interchange of transmitter and receiver boreholes instead of a one-sided equidistant setup in either borehole was proposed and yielded promising results [25]. They concluded that the use of the semi-reciprocal setup is optimum for acquisition speed, inversion speed. Gueting et al. [26] demonstrated the value of using FWI of cross-hole radar data for aquifer characterization and facies analysis. They concluded that FWI of cross-hole radar data followed by cluster analysis is an applicable approach to identify hydrogeological facies in alluvial aquifers and to map their architecture and connectivity.

In this paper, we deduce the gradient formula of the objective function with respect to both permittivity and conductivity by deducing derivative directly other than perturbation method [17], obtain the same gradient formulae as previous work [17] and realize the simultaneous inversion of permittivity and conductivity using a conjugate gradient method. In the process of inversion, forward modeling is realized by solving the Maxwell’s equation using two-dimensional second-order time domain finite difference method (FDTD) based on the Complex Perfected Matched Layer (CPML) absorbing boundary condition [27].

We first test the inversion algorithm using two synthetic examples and find that the FWI can provide high-resolution imaging of permittivity and conductivity; the targets as small as one-half to one-third wavelength can be distinguished. Then we apply the FWI algorithm to a field cross-hole radar data in order to evaluate the stability of subsurface foundation. We find many anomalies which are interpreted as subsurface mined-out zones.

## 2. Theory of FWI

We realize the FWI inversion algorithm for cross-hole radar data. The main purpose of the FWI is to invert the spatial distribution of both the permittivity ε and conductivity σ by minimizing the objective function [17]:

(1) $$S(\varepsilon ,\sigma )=\frac{1}{2}\sum_{s}\sum_{d}\sum_{\tau }{\left[E^{s}(\varepsilon ,\sigma )-E_{obs}^{s}\right]}_{d,\tau }^{T}\cdot {\left[E^{s}(\varepsilon ,\sigma )-E_{obs}^{s}\right]}_{d,\tau }\;$$

where the $E^{s}(\varepsilon ,\sigma )$ and $E_{obs}^{s}$ are the forward and observed data at the receivers, respectively. The superscript “s” stands for a particular source. Equation (1) is the sum of the difference between the forward and observed data in three ranges including the source position *s*, receiving position *d* and the observation time τ.

We try to obtain the gradient (see Appendix A) by deducing the derivation of the objective function. The gradient at each spatial position can be expressed as [17],

(2) $$\left[\begin{array}{l} \nabla S_{\varepsilon }\\ \nabla S_{\sigma } \end{array}\right]=\sum_{s}\sum_{\tau }\begin{array}{c} {\left(\partial_{t}E^{s}\right)}^{T}\left({\hat{G}}^{T}R^{s}\right)\\ {\left(E^{s}\right)}^{T}\left({\hat{G}}^{T}R^{s}\right) \end{array}\;$$

where, ${\hat{G}}^{T}$ represents backward propagated process of the sum of residual $R^{s}$, ${\hat{G}}^{T}R^{s}$ can be interpreted as the backward propagated process of the sum of residual, and

(3) $$R^{s}=\sum_{d}\left(E^{s}-E_{obs}^{s}\right)\;$$

In order to increase the rate of convergence, we use the conjugate gradient method [28] to update the model parameters:

(4) $$\left[\varepsilon {\left(x\right)}_{k+1}\right]=\left[\varepsilon {\left(x\right)}_{k}\right]-\zeta_{\varepsilon ,k}\cdot \left[C_{\varepsilon }{\left(x\right)}_{k}\right]\;$$

(5) $$\left[\sigma {\left(x\right)}_{k+1}\right]=\left[\sigma {\left(x\right)}_{k}\right]-\zeta_{\sigma ,k}\cdot \left[C_{\sigma }{\left(x\right)}_{k}\right]\;$$

where, $\zeta_{\varepsilon ,k}$ and $\zeta_{\sigma ,k}$ are the iterative step increments of the permittivity and conductivity at the *k*-th iteration, respectively. $C_{\varepsilon }{\left(x\right)}_{k}$ and $C_{\sigma }{\left(x\right)}_{k}$ are the conjugate gradients for the permittivity and conductivity, respectively,

(6) $$C_{\varepsilon }{\left(x\right)}_{k}=\nabla S_{\varepsilon }{\left(x\right)}_{k}+\frac{\nabla S_{\varepsilon }{\left(x\right)}_{k}\left(\nabla S_{\varepsilon }{\left(x\right)}_{k}-\nabla S_{\varepsilon }{\left(x\right)}_{k-1}\right)}{\nabla S_{\varepsilon }{\left(x\right)}_{k-1}\nabla S_{\varepsilon }{\left(x\right)}_{k-1}}C_{\varepsilon }{\left(x\right)}_{k-1}\;$$

(7) $$C_{\sigma }{\left(x\right)}_{k}=\nabla S_{\sigma }{\left(x\right)}_{k}+\frac{\nabla S_{\sigma }{\left(x\right)}_{k}\left(\nabla S_{\sigma }{\left(x\right)}_{k}-\nabla S_{\sigma }{\left(x\right)}_{k-1}\right)}{\nabla S_{\sigma }{\left(x\right)}_{k-1}\nabla S_{\sigma }{\left(x\right)}_{k-1}}C_{\sigma }{\left(x\right)}_{k-1}\;$$

As *k* = 1, $C_{\varepsilon }{\left(x\right)}_{1}=\nabla S_{\varepsilon }{\left(x\right)}_{1}$ and $C_{\sigma }{\left(x\right)}_{1}=\nabla S_{\sigma }{\left(x\right)}_{1}$. By searching for the extreme points along the $C_{\varepsilon }$ and $C_{\sigma }$ directions, we can obtain the simultaneous inversions of the permittivity and conductivity at the same iteration.

The iteration step increments are obtained by the following formulae [17,29],

(8) $$\zeta_{\varepsilon ,k}=\kappa_{\varepsilon }\frac{\sum {}_{s}\sum {}_{d}\sum {}_{\tau }{\left[E^{s}(\varepsilon +\kappa_{\varepsilon }C_{\varepsilon ,k},\sigma )-E^{s}(\varepsilon ,\sigma )\right]}_{d,\tau }^{T}{\left[E^{s}(\varepsilon ,\sigma )-E_{obs}^{s}\right]}_{d,\tau }}{\sum {}_{s}\sum {}_{d}\sum {}_{\tau }{\left[E^{s}(\varepsilon +\kappa_{\varepsilon }C_{\varepsilon ,k},\sigma )-E^{s}(\varepsilon ,\sigma )\right]}_{d,\tau }^{T}{\left[E^{s}(\varepsilon +\kappa_{\varepsilon }C_{\varepsilon ,k},\sigma )-E^{s}(\varepsilon ,\sigma )\right]}_{d,\tau }}\;$$

(9) $$\zeta_{\sigma ,k}=\kappa_{\sigma }\frac{\sum {}_{s}\sum {}_{d}\sum {}_{\tau }{\left[E^{s}(\varepsilon ,\sigma +\kappa_{\sigma }C_{\sigma ,k})-E^{s}(\varepsilon ,\sigma )\right]}_{d,\tau }^{T}{\left[{Ε}^{s}(\varepsilon ,\sigma )-E_{obs}^{s}\right]}_{d,\tau }}{\sum {}_{s}\sum {}_{d}\sum {}_{\tau }{\left[E^{s}(\varepsilon ,\sigma +\kappa_{\sigma }C_{\sigma ,k})-E^{s}(\varepsilon ,\sigma )\right]}_{d,\tau }^{T}{\left[E^{s}(\varepsilon ,\sigma +\kappa_{\sigma }C_{\sigma ,k})-E^{s}(\varepsilon ,\sigma )\right]}_{d,\tau }}\;$$

Here, $\kappa_{\varepsilon }$ and $\kappa_{\sigma }$ are stability factors, which should be carefully selected in the inversion.

## 3. Synthetic Examples

We first use two synthetic examples to check the performance of FWI, then we move to a field data set for mined-out zone detection and evaluation.

### 3.1. Synthetic Data 1: A Simple Model

**Figure 1:**
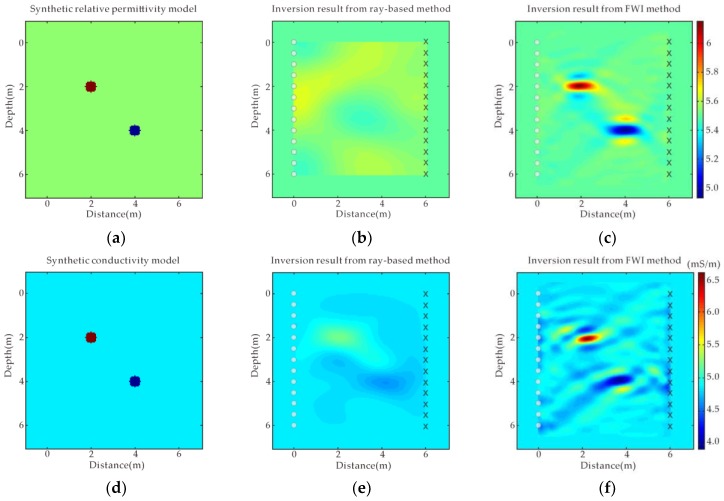
A simple synthetic model with two pipeline bodies buried in homogeneous medium. The model parameters of (**a**) relative permittivity and (**d**) conductivity; ray-based results of (**b**) relative permittivity and (**e**) conductivity; full waveform inversion (FWI) results of (**c**) relative permittivity and (**f**) conductivity.

**Figure 2:**
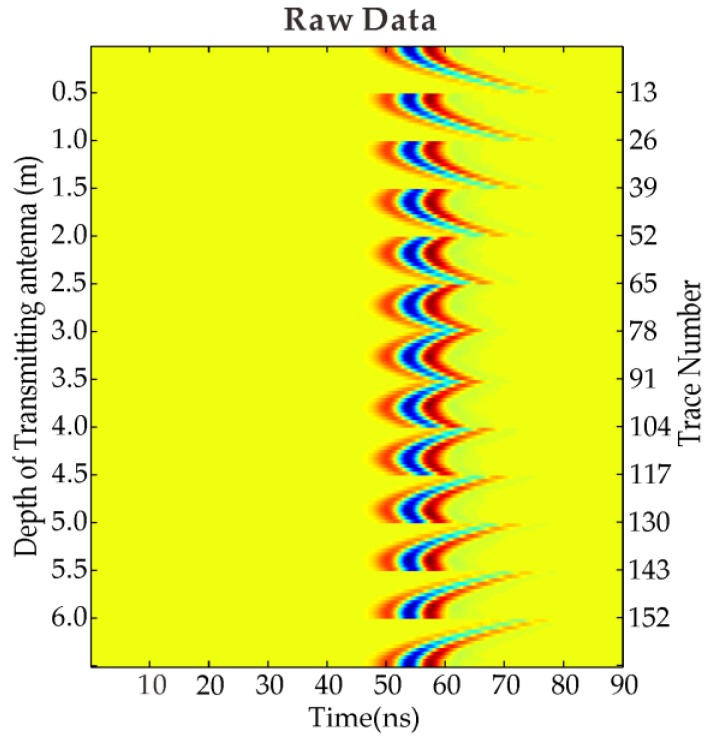
Raw data of simple synthetic model with two pipeline bodies buried in homogeneous medium.

We create a 2D homogeneous model as shown in Figure 1a (for permittivity) and Figure 1d (for conductivity) in order to verify the performance of our time domain FWI algorithm. The model size is 6 m in both x- and y-directions. There are two pipeline anomalies buried in the homogeneous background medium with a diameter of 0.5 m. The relative permittivity and conductivity of the left upper anomaly located at the position of (2 m, 2 m) are 7 and 0.008 S/m, respectively; while the relative permittivity and conductivity of the right lower anomaly located at the position of (4 m, 4 m) are 4 and 0.003 S/m, respectively. The relative permittivity and conductivity of the background medium are 5.5 and 0.005 S/m, respectively. A total of 13 transmitter positions (circles indicated) and 13 receiver positions (crosses indicated) with 0.5 m spacing interval are included in this example. The initial permittivity and conductivity of the model are the same as the background medium with the relative permittivity and conductivity of 5.5 and 0.005 S/m. Figure 2 shows the raw data of simple synthetic model with two pipeline bodies buried in a homogeneous medium. A Ricker wavelet with the center frequency of 100 MHz is employed in this example, therefore the source wavelet is known in the inversion process. The grid size is 0.05 m; the iteration number is 50 for the inversion.

In order to hold the linear approximation, the choice of the stability factor follows such two different rules [17,29]: (1) it has to be small enough so that the perturbed model still lies in the linearity range; (2) it has to be large enough to avoid truncation (round-off) errors when dealing with small numbers in the computer. Stability factors $\kappa_{\varepsilon }$ and $\kappa_{\sigma }$ are chosen according to the value of the gradients here. As the product of the step increment and the maximum gradient less than the order of magnitude of the model parameter (conductivity or permittivity), the selections of factors $\kappa_{\varepsilon }$ and $\kappa_{\sigma }$ are needed. After numerous tests, $\kappa_{\varepsilon }$ and $\kappa_{\sigma }$ are chosen to be 10^−8^ and 100 in our inversion, respectively.

In the process of calculating the gradient, we need to calculate the forward and backward propagated fields. In the process of forward modeling, there is a strong near-field effect which will adversely influence the inversion results. In this paper, a median filter is utilized to eliminate the near-field effect, which can effectively suppress the interference of the source and will not change the overall gradient. Median filtering is a nonlinear smoothing technique, which sets the value of each pixel with the median value of all the pixels within the filter window [30]. In addition, the permittivity and conductivity are transformed to logarithms in the inversion process to keep the inverted values physically positive and to improve the rate of convergence and stability of FWI at the same time.

**Figure 3:**
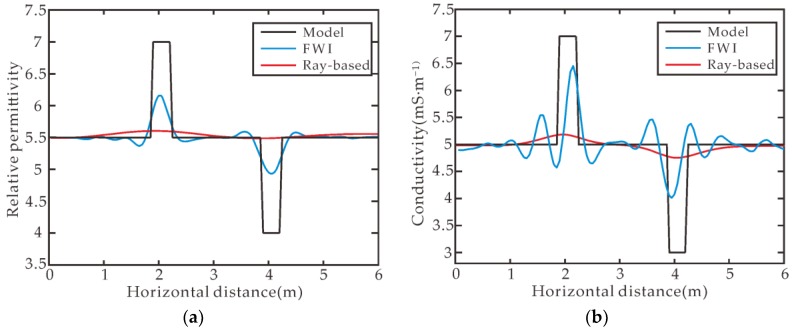
Comparisons among the inverted results from FWI, ray-based, and the true model: (**a**) relative permittivity and (**b**) conductivity along the line from the 1st transmitter position to the 13th receiver position.

Figure 1b,e shows the inverted relative permittivity and conductivity from the ray-based method [5,31], respectively, from which two pipeline anomalies cannot be distinguished clearly. Figure 1f shows the FWI inverted relative permittivity and conductivity from which we can find that the FWI inverted results agree well with the model values, and the image of these two anomalies is clearly depicted with correct position and numerical values. Figure 3a,b shows the sections of the relative permittivity and conductivity through the transmitter position #1 and the receiver position #13 by FWI, and ray-based method, respectively. The positions and sizes of the anomalies are well depicted in FWI inversion results, but not in ray-based results. Comparing the FWI results with the model values, the peak value of the inverted conductivity comes closer to the model value than permittivity’s case but there is more wavy interference around the peak than the case for permittivity. For the first pipeline anomaly with conductivity of 0.007 S/m, the FWI inverted conductivity peak value can reach 0.0065 S/m whereas the ray-based method inverted conductivity only reaches 0.0052 S/m. Ernst et al. got similar results [16]. GPR is more fitted in the low-loss case than in the lossy case generally, however, the radar wave is sensitive to both the permittivity and the conductivity. The synthetic example proves the effectiveness of the FWI in target positioning, physical values estimation and target geometry delineation.

### 3.2. Synthetic Data 2: A Complex Model

**Figure 4:**
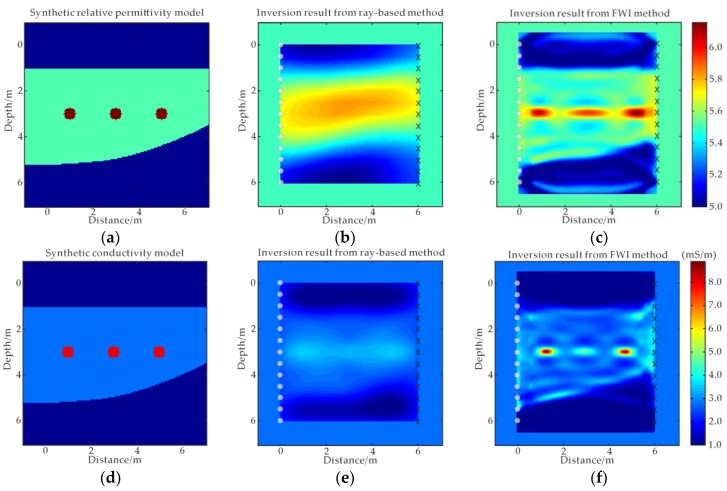
A complex synthetic model with three pipeline anomalies buried in the layered medium. Model parameters of (**a**) relative permittivity and (**d**) conductivity; ray-based results of (**b**) relative permittivity and (**e**) conductivity; FWI results of (**c**) relative permittivity and (**f**) conductivity.

**Figure 5:**
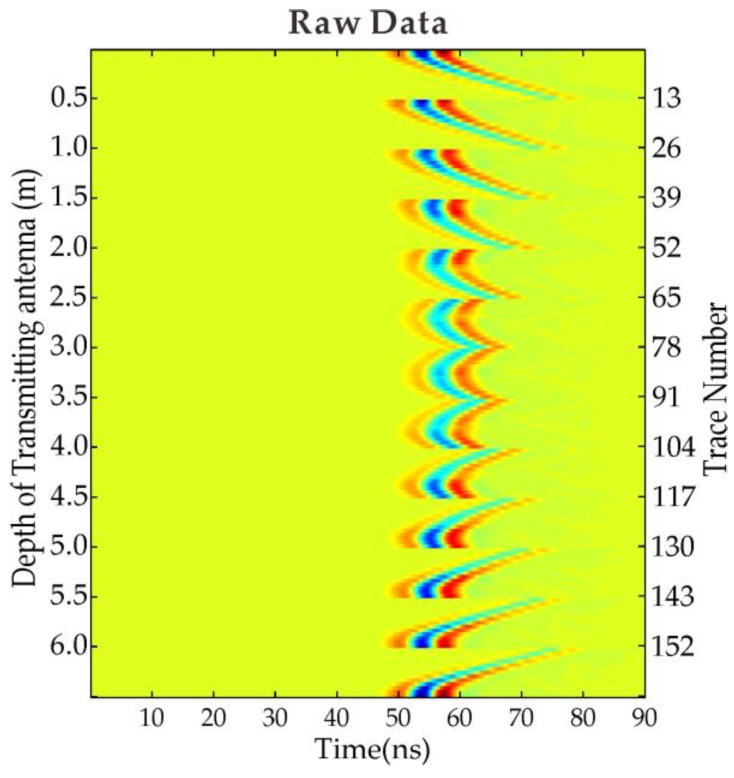
Raw data of complex synthetic model with three pipeline anomalies buried in the layered medium.

In order to further verify the performance of FWI in the time domain, a layered model with the same size as the first example is built as shown in Figure 4a,d. The model contains three pipeline anomalies with diameter of 0.5 m, buried in the three-layered medium. The relative permittivity and conductivity of the three anomalies located at the position of (1 m, 3 m), (3 m, 3 m) and (5 m, 3 m) are 7 and 0.008 S/m, respectively. The relative permittivity and conductivity of the top- and the bottom-layer medium are both 5 and 0.001 S/m, respectively, while they become 5.5 and 0.0028 S/m, respectively, for the mid-layer medium. The initial permittivity and conductivity values are the same as the mid-layer medium. Other parameters are the same as the first example. Figure 5 shows the raw data of complex synthetic model with three pipeline anomalies buried in the layered medium.

**Figure 6:**
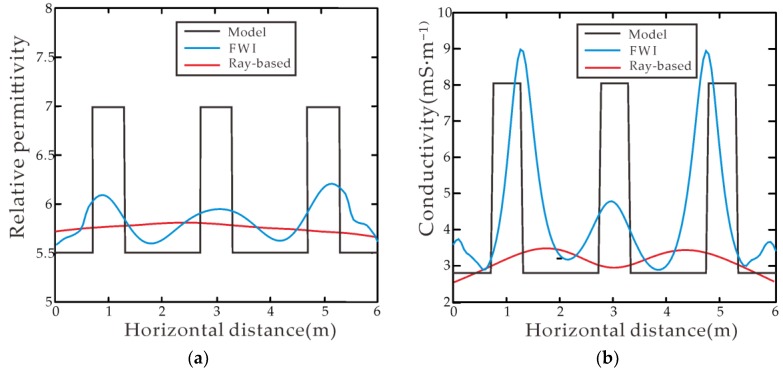
Comparisons among the inverted results from FWI, ray-based, and the true model at the depth of 3 m: (**a**) relative permittivity and (**b**) conductivity.

Figure 4b,e shows the inverted relative permittivity and conductivity result from the ray-based method [5,31], respectively, from which three pipelines cannot be distinguished, although the layered medium can be delineated roughly. From the FWI inverted permittivity and conductivity as shown in Figure 4c,f, respectively, one can note that the top and bottom layers can correctly be reconstructed, the inversion results agree well with the model, and the image of these three anomalies is clearly depicted with correct positions and numerical values. Figure 6a,b shows sections of the relative permittivity and the conductivity at the depth of 3 m by FWI, and by ray-based method, respectively. Comparing the inverted values of relative permittivity and conductivity by ray-based method with those obtained from FWI, one can see that FWI provides the better performance than the ray-based method.

According to previous publications, the resolution for the ray-based method is about one wavelength [7] while the resolution for FWI is from one-half to one-third wavelength [32,33]. The high-resolution of FWI has been proven by the synthetic examples. The pipeline anomalies with 0.5 m diameter can be distinguished as the wavelength of the central frequency 100 MHz is about 1.28 m. Even in the layered medium, targets can be positioned and delineated with high-resolution.

## 4. Field Data Measured at Xiuyan Jade Mine

**Figure 7:**
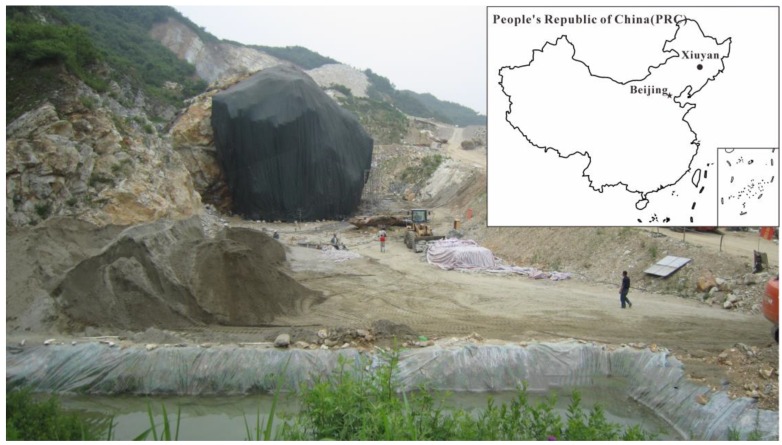
Field survey site scene and it location map in China. The giant jade is at the center of the photo.

**Figure 8:**
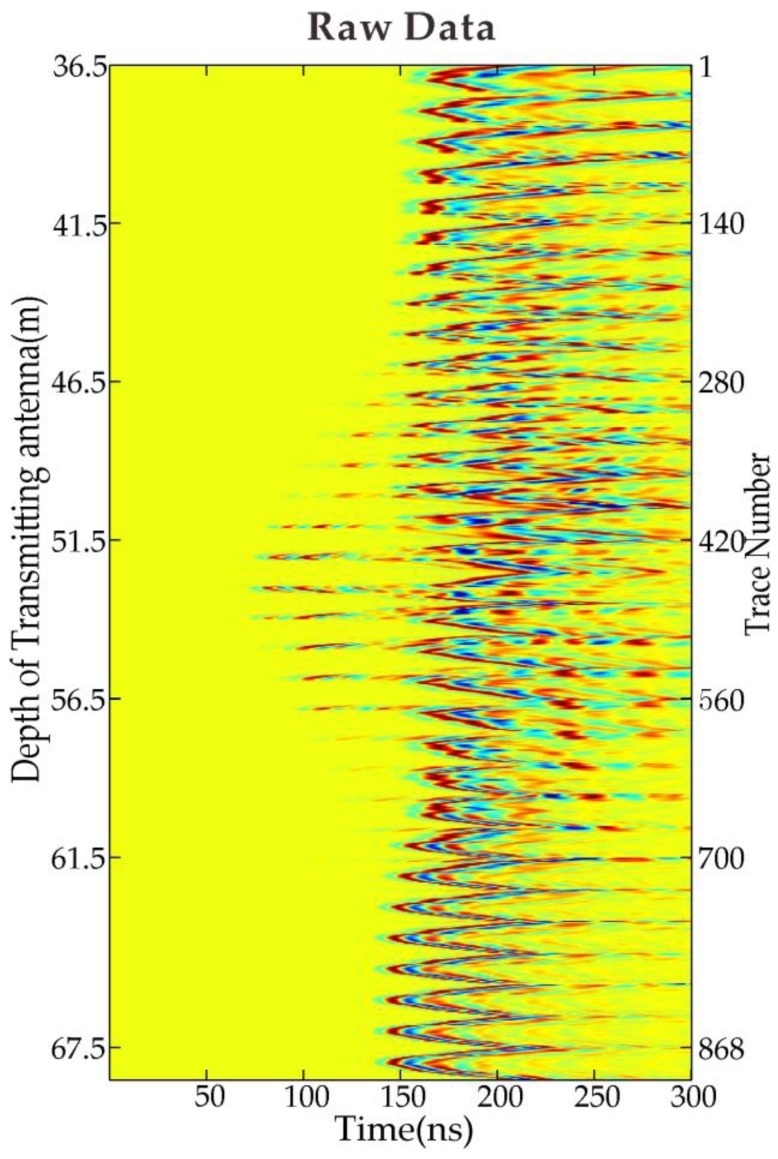
Measured cross-hole radar raw data at Xiuyan mine.

Xiuyan Jade, produced in Xiuyan County, Liaoning Province, China is one of the four famous jade in China. King Jade, which is deemed to be the largest jade body of the world, was a broken jade body from a hill (see Figure 7). The local government planned to build a tourism site based on the jade culture there. The investigation site was located at the foot of King Jade. The purpose of the investigation was to evaluate the stability of subsurface foundation, to find the possible positions of the mined-out zone, to figure out filling conditions of the subsurface void space, and to prevent the further collapse and rolling of the jade body. We used a self-developed borehole radar system based on Vector Network Analyzer (VNA) [34] to perform the measurement. The central frequency of the antenna was around 100 MHz during the data acquisition. The horizontal distance between the two vertical boreholes was 16 m. Both boreholes were more than 100 m deep originally but became shallower because of the falling material filling at the bottom. The upper parts of both boreholes were cased with metal pipes. Therefore, the detectable range by radar is shortened. There are a total of 32 source positions with 1 m intervals evenly distributed from the depth of 36.5 m to 67.5 m. Each source position corresponded to a total of 28 receiver positions with 1 m interval. For the first source at 36.5 m, the receiver started from 23.5 m and moved downward till 50.5 m. As the source position move 1 m downwards, the receiver scan range moved 1m downward also. Figure 8 shows the raw data measured at the site.

Knowledge of source wavelet is crucial for FWI in the field data case because we must know the source wavelet in forward modeling, but in fact we do not know it. Therefore, an especially difficult method is adopted by following reference [18] to extract the source wavelet. The basic principle is summarized below. A cross-hole radar measurement can be effectively approximated as the convolution of the earth’s impulse response with the radar source wavelet in the absence of pronounced nonlinear phenomena. As a result, deconvolving this impulse response from the measured data should yield an estimate of the source wavelet and performing the deconvolution using a number of different measurements should increase the reliability of the estimation. The estimation processing is carried out in the frequency domain. The true impulse response of the Earth is a priori unknown, but a reasonable approximation can be obtained by forward modeling through an approximate subsurface property model, such as that obtained using standard ray-based tomography for the first time estimation updated model for the later time estimation. In other words, we can first calculate synthetic radar data through our best guess of the subsurface model using a known initial source wavelet and then deconvolve this known wavelet from the synthetic data in order to obtain an estimate of the earth’s impulse response. The next step is to estimate the unknown field source wavelet by deconvolving the impulse response of the earth from the observed field radar data. In principle, the earth’s impulse response and the corresponding estimation of the source wavelet need to be evaluated separately for each transmitter-receiver configuration considered. Nonetheless, in practice, we use a single average source wavelet from the parallel measurements. Once the source wavelet has been estimated, the previously described waveform inversion algorithm can be run until convergence is achieved. If eventual convergence seems unlikely after a certain number of iterations, then a new source wavelet may be required.

Before starting the FWI, a forward modeling is performed to estimate the initial source wavelet which is obtained by the deconvolution method based on the initial model. In the subsequent process of the inversion, we carry out two times of source wavelet estimation after 15 times and 28 times iterations, respectively. The total iteration number is 34 in the inversion. FWI calculation is carried out with a computer cluster including two PCs, each of which is installed with a 6-core CPUs. The process of the inversion iteration lasted for about 4 h, so the FWI is still very time-consuming. All region between two holes and from depth of 23.5 m to 82 m is discretized with 0.05 m grid in both horizontal and vertical directions for forward modelling and inversion.

**Figure 9:**
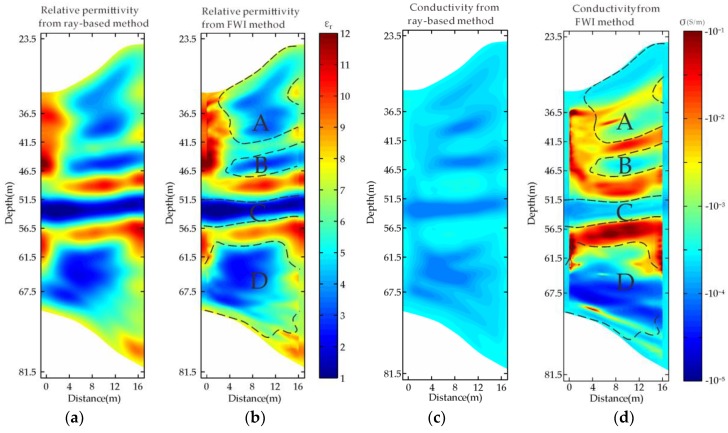
Inverted results of Xiuyan field data: ray-based results of (**a**) relative permittivity and (**c**) conductivity; FWI results of (**b**) relative permittivity and (**d**) conductivity.

Figure 9a,c shows initial models, which are obtained from the ray-based method [5,30]. From Figure 9a,b, we find that the details of the estimated relative permittivity from the FWI have been enriched compared to that from the travel time tomography, for example, at the right-bottom corner. From Figure 9c,d, the conductivity obtained from the FWI is significantly improved from the initial model in both imaging contrast and details. At the depth range from 36.5 m to 46.5 m, the conductivity distribution conforms with that of the permittivity.

**Figure 10:**
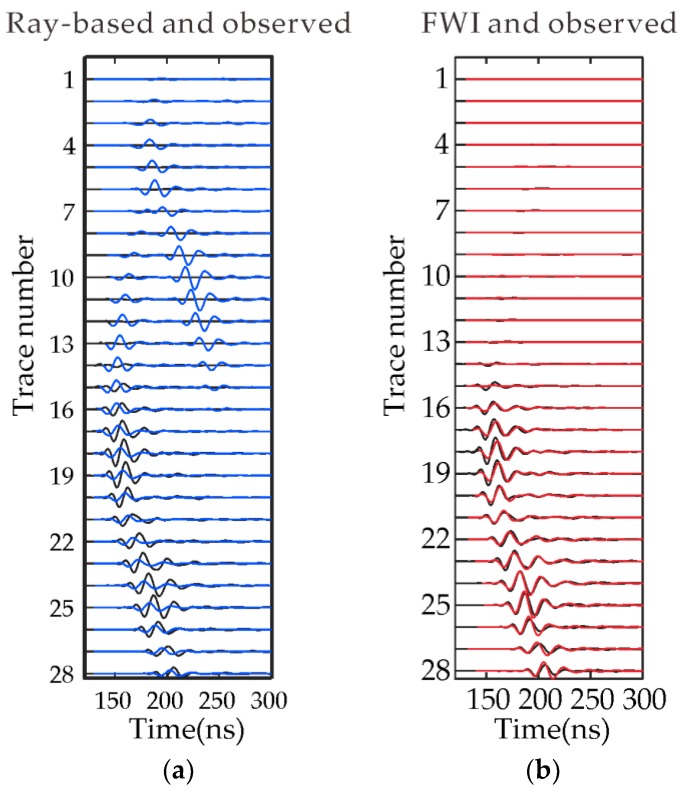
Waveform comparisons for receiver gathers when the source is at the depth of 63.5 m: (**a**) The solid and dashed lines show the observed radar traces and data generated from the ray-based results; (**b**) the solid and dotted lines show the observed radar traces and data from the FWI results. Amplitudes in all panels are normalized with respect to the maximum amplitude of the field data.

Figure 10 shows the waveform comparison between the observed, the forward data based on the FWI and ray-based results for the 28th source position. Figure 10a shows the comparison between the forward waveforms from ray-based results and the field measured waveforms, it can be clearly seen that there are still smaller differences between the phases, however, the forward waveforms from the FWI results as shown in Figure 10b are obviously in better agreement with the field measured waveforms. The residual error from FWI results is smaller than that from the ray-based results. The relative errors are 6.82% and 129.32% for the FWI and ray-based results, respectively. The relative error for the ray-based result is so high because the ray-based method only matches the arrival time or the amplitude of the first cycle. Waveforms comparison proves the accuracy of FWI.

**Figure 11:**
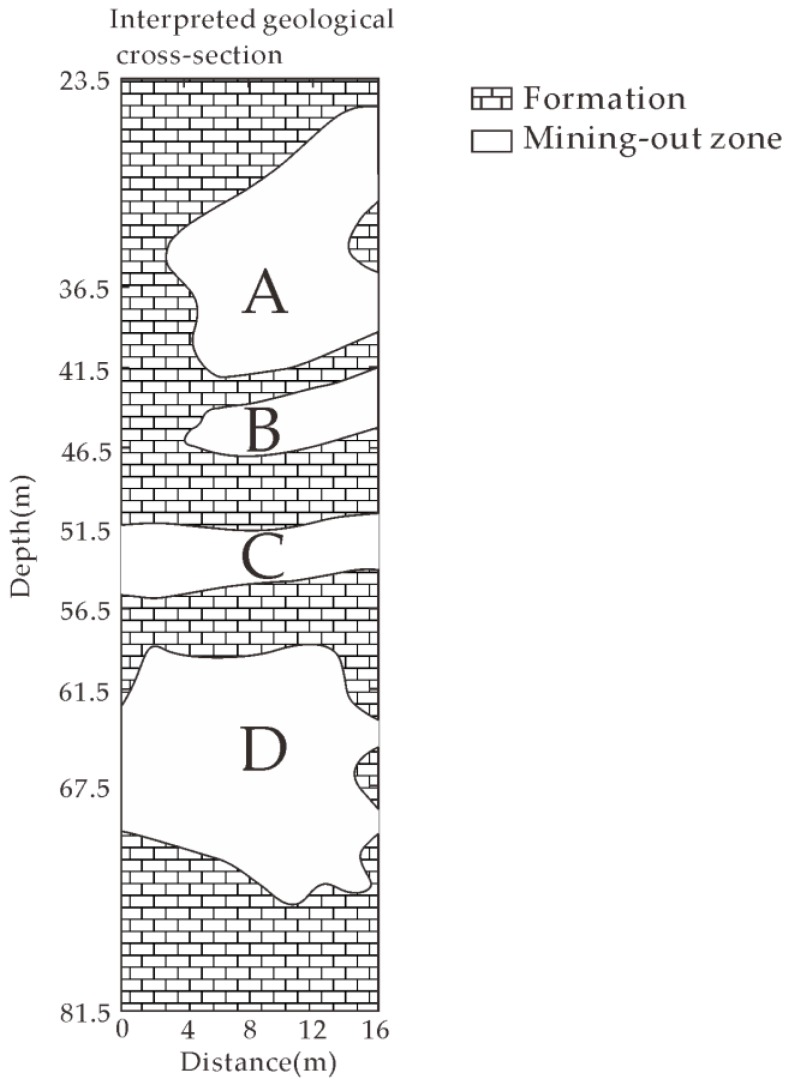
Interpreted geological cross-section from inverted results between two boreholes.

According to the existing mining and geological data, we know that there are five mining tunnels within 100 m depth range beneath the giant jade. Mining activity was carried out along these channels and meanwhile various mined-out zones were formed. There are possibly different filling situations in these mined-out zones, such as the collapse, cement grouting, void and so on. According to the known subsurface information and the FWI inverted permittivity and conductivity results, we interpret the subsurface situation. We found from Figure 9 that there were two low-permittivity and low-conductivity zones in the range from 36.5 m to 46.5 m marked by “A” and “B”, separated by a banded anomaly whose permittivity and conductivity values are between that of the air and rock. We interpret that they are resulted from two mining tunnels related to mining activity in the upper part. There is a narrow mined-out zone at the depth range from 51.5 m to 56.5 m marked by “C” whose permittivity and conductivity are close to that of the air, and it exhibits a mining tunnel characteristic. There is a larger mining zone marked by “D” which is possibly resulted from two mined-out tunnels connecting together due to the collapse at the depth from 61.5 m to 67.5 m, whose permittivity and conductivity values are between that of the air and rock. Therefore, the interpreted mined-out zones are all resulted from the five mining tunnels as documented according to our understanding. The detected mined-out zones exhibit low-permittivity and low-conductivity characteristics therefore they are all empty and not water-filled. Only small value difference exists in the inverted values for these four mining-out zones. Two reasons could lead to this difference. Firstly, the filling conditions are slightly different for different mining-out zones. Secondly, the FWI could have errors which lead to the difference. However, the FWI based cross-hole radar method is appropriate for high contrast geological environment. According to the above analysis, the interpreted geological structure model is sketched as shown in Figure 11.

## 5. Discussion

Usually the local minima often exist in nonlinear inversion, while the purpose of the inversion is to find the global minima. The gradient-based inversion always has this kind of problem, while Monte Carlo inversion, such as simulated annealing, genetic algorithms, and neighborhood algorithm [35,36,37,38], may overcome it. The popular inversion method in geophysics is still gradient-based currently because Monte Carlo inversion is too computationally heavy when dealing with geophysical problems with big data. Therefore, the estimation of the initial model is an important step to overcome the local minima problem. The ray-based method provides a relatively accurate initial model for inversion. Sometimes, geophysical inversion is constrained by prior information.

Considering the general applicability of the method, the background knowledge is necessary for geophysical interpretation, such as the data from general geology, drilling, engineering geology, hydrological geology etc. The main reason is that geophysical problems have the nature of multiple solutions. Only integrating different knowledge can overcome the non-uniqueness and improve the accuracy, credibility of the geophysical problem. Therefore, the method is applicable in a wide range of applications from the sense of geophysical prospecting.

## 6. Conclusions

The results of a cross-hole radar survey at Xiuyan Jade mine, Liaoning province, China, were presented. The survey was carried out using a self-developed VNA-based radar system. The ray tracing tomography and the FWI inversion method were used to invert a measured data set comparably. The superiority of the FWI over the ray tracing method to detect the pipeline targets was investigated using synthetic models. It is shown that FWI can locate the sub-wavelength targets, invert the physical values of targets and delineate the layer boundary accurately, however, ray tracing method cannot. The FWI results resolve four subsurface mined-out zones more clearly than the ray tracing method especially in the conductivity tomography. The mined-out zones are characterized by low-permittivity and low-conductivity in the FWI results. Both the permittivity and the conductivity from FWI can map the mined-out zones comparably. The interpreted mined-out zones are in accordance with existing five mining channels recorded in geological data. FWI based tomography for cross-hole radar measurement is thus a powerful tool for subsurface mined-out zones detection and evaluation.

## Appendix A

Omitting the summation in three ranges for clear expression, the objective function can be simplified as:

(A1) $$S(\varepsilon ,\sigma )=\frac{1}{2}{\left[E^{s}(\varepsilon ,\sigma )-E_{obs}^{s}\right]}_{d,\tau }^{T}\cdot {\left[E^{s}(\varepsilon ,\sigma )-E_{obs}^{s}\right]}_{d,\tau }$$

The gradient corresponding to the objective function is obtained by the derivation of (A1) with respect to the model parameters. For simplicity, we use *p* to represent model parameters ε and σ,

(A2) $$\frac{\partial S}{\partial p}=\frac{\partial E^{s}}{\partial p}\cdot {\left(E^{s}-E_{obs}^{s}\right)}_{d,\tau }$$

where $\frac{\partial E^{s}}{\partial p}$ is unknown that cannot be obtained directly from the objective function. The right side includes two terms: partial directive e$\frac{\partial E^{s}}{\partial p}$ and residual source.

In the time domain, the Maxwell equations can be expressed as [17],

(A3) $$\left[\begin{array}{cc} -\varepsilon (x)\partial_{t}-\sigma (x) & \nabla \times \\ \nabla \times & \mu_{0}\partial_{t} \end{array}\right]\left[\begin{array}{l} E^{s}(x,t)\\ H^{s}(x,t) \end{array}\right]=\left[\begin{array}{l} J^{s}(x,t)\\ 0 \end{array}\right]$$

Equation (A3) is abbreviated as follows,

(A4) $$M(\varepsilon ,\sigma )\left[\begin{array}{l} E^{s}\\ H^{s} \end{array}\right]=\left[\begin{array}{l} J^{s}\\ 0 \end{array}\right]$$

where **M** is a linear operator that represents Maxwell’s equation. If ignoring the magnetic field, (A4) becomes,

(A5) $$ME^{s}=J^{s}$$

We have $E^{s}=M^{-1}J^{s}$. In the time domain, the electric field of arbitrary position can be considered as the convolution between the source wavelet and **G**

(A6) $$E^{s}=G*J^{s},\mathrm{therefore}\;G=M^{-1}$$

where $G=\left[g_{x,1},g_{x,2},\cdots g_{x,nr}\right]$ is the Green’s operator of **M**, and *nr* is the number of receivers [11]. Taking the partial derivative of both sides of (A5) with respect to the parameter *p*, we can obtain

(A7) $$\frac{\partial M}{\partial p}{Ε}^{s}+M\frac{\partial {Ε}^{s}}{\partial p}=0,\mathrm{and}\;\frac{\partial {Ε}^{s}}{\partial p}=M^{-1}v$$

where we have introduced the virtual source term (A8),

(A8) $$v=-\frac{\partial M}{\partial p}E^{s}$$

Within the **M** in (A3), only term $-\varepsilon (x)\partial_{t}-\sigma (x)$ has relationship with the model parameters ε and σ. Therefore,

(A9) $$\{\begin{array}{c} v_{\varepsilon }=-\frac{\partial M}{\partial \varepsilon }{Ε}^{s}=\partial_{t}E^{s}\\ v_{\sigma }=-\frac{\partial M}{\partial \sigma }E^{s}=E^{s} \end{array}$$

Substituting (A7) into (A2) and rearranging it, it leads to

(A10) $$\frac{\partial S}{\partial p}=[M^{-1}v]\cdot {\left(E^{s}-E_{obs}^{s}\right)}_{d,\tau }$$

According to (A6), (A10) can be rewritten as green’s function format as,

(A11) $$\frac{\partial S}{\partial p}=[G*v]\cdot {\left(E^{s}-E_{obs}^{s}\right)}_{d,\tau }$$

Because the order exchange of two items within a convolution will not affect the result, (A11) becomes

(A12) $$\frac{\partial S}{\partial p}=[v*G]\cdot {\left(E^{s}-E_{obs}^{s}\right)}_{d,\tau }$$

Rewriting (A12) into an integral form, and including the summation in three ranges, it becomes

(A13) $$\frac{\partial S}{\partial p}=\sum_{i=1}^{ns}\sum_{j=1}^{nr}\int_{-\infty }^{\infty }\left[\int_{-\infty }^{\infty }v_{i,x}\left(\xi -\tau \right)g_{x,j}\left(\tau \right)d\tau \right]r_{ij}\left(\xi \right)d\xi$$

where *ns* and *nt* are the number of transmitters and receivers, and residual source $r_{ij}\left(\xi \right)=E\left(\xi \right)-E^{obs}\left(\xi \right)$.

It is noted that $v_{i,x}\left(\xi -\tau \right)$ and $g_{x,j}\left(\tau \right)$ have different subscript, because virtual resource $v_{i,x}\left(\xi -\tau \right)$ corresponds to transmitter while the green function $g_{x,j}\left(\tau \right)$ corresponds to the receiver. Let ξ−τ=t, we obtain dτ=−dt. Rearranging (A13), we obtain

(A14) $$\frac{\partial S}{\partial p}=\sum_{i=1}^{ns}\sum_{j=1}^{nr}\int_{-\infty }^{\infty }v_{i,x}\left(t\right)\left[\int_{-\infty }^{\infty }g_{x,j}\left(\xi -t\right)r_{i,j}\left(\xi \right)d\xi \right]dt$$

(A15) $$\frac{\partial S}{\partial p}=\sum_{i=1}^{ns}\sum_{j=1}^{nr}\sum_{t=1}^{nt}v\left(Gr_{ij}\right)\;$$

where *nt* is the time window length. According to (A15), the gradient can be expressed as gradient Equation (2).
